# Supplementary material for: Latent space-based network analysis for brain–behavior linking in neuroimaging
Source: Nat Methods. 2025 Dec 4;23(1):225–35. doi: 10.1038/s41592-025-02896-9 (PMC13002467; doi:10.1038/s41592-025-02896-9)
Supplement: Supplementary file 1 — Supplementary Figs. 1–8 and Tables 1–3. [file 41592_2025_2896_MOESM1_ESM.pdf]

nature methods

Supplementary information

https://doi.org/10.1038/s41592-025-02896-9

Latent space-based network analysis for brain–behavior linking in neuroimaging

In the format provided by the authors and unedited

Additional Simulation Results

We show the barplots comparing the power (A) and specificity (B) of LatentSNA with CPM, Lasso and CCA in different data situations of the simulation (Supplementary Figure 1). From left to right, the sample size increases from 500, 1000 to 2000. From top to bottom, we include small ($V = 20$) and large ($V = 70$) networks, as well as relatively small (0.5) and large (1) signal to noise ratios. We show the correlations between the predicted and observed behavior across 100 replications under different simulation conditions (Supplementary Figure 2). We show the correlations between the predicted and observed connectivity across 100 replications (Supplementary Figure 3). Given that the Average method use the population-level estimates to predict individual connectivity in the test data, we use population-level connectivity in addition to the individual-level connectivity estimates to perform prediction. Real-world functional brain connectivity tends to have large correlations with the average connectivity, and therefore, we also test the prediction performance of the average latent connectivity estimates.

Additional Application Results

We show scatter plots of the observed internalizing values against the connectivity of 5 internalizing regions and 5 non-internalizing regions (region indices are shown at the top left corner in black) identified by the model (Supplementary Figure 4). The first and third columns show the scatter plots of the observed internalizing values against the estimated latent connectivity using LatentSNA. The second and forth columns show the scatter plots of the corresponding CPM for a specific brain region, where the predictors are the sums of the significant connectivity edges based on Pearson correlations. We show the location and connectivity networks of the top 5 internalizing regions with the strongest relationships with internalizing and non-internalizing regions with no identifiable relationships with internalizing (Supplementary Figure 5). The circle plots color groups according to anatomical locations, and the 3D brain plots show the front (top left), back (top right), right (bottom left) and left (bottom right) views. We show the histograms of the estimated covariances between functional connectivity networks and internalizing psychopathology in the 4 conditions (Supplementary Figure 6).

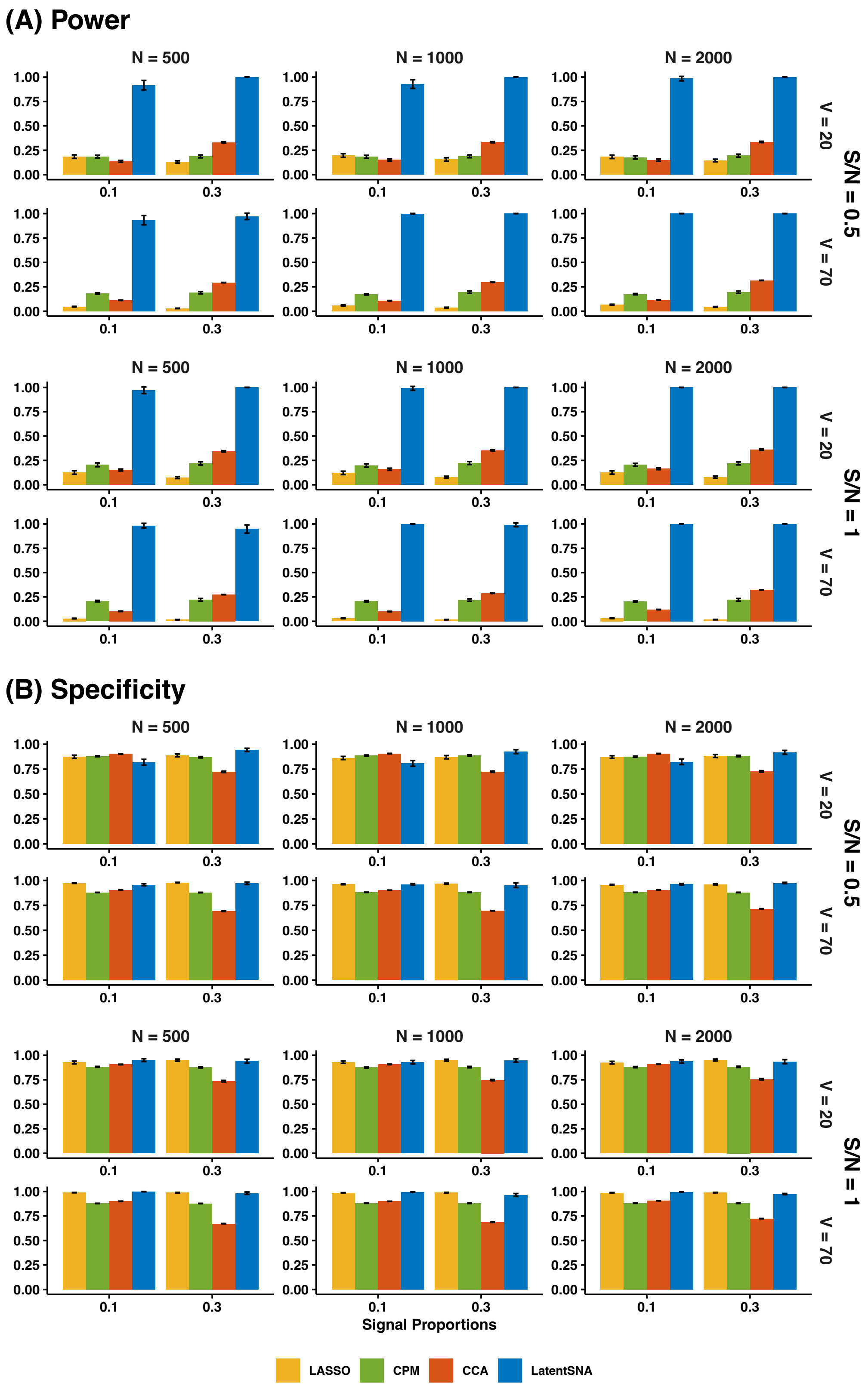

Supplementary Figure 1: The barplots comparing the power (A) and specificity (B) of LatentSNA with CPM, Lasso and CCA in different data situations. From left to right, the sample size increases from 500, 1000 to 2000. From top to bottom, we include small ($V = 20$) and large ($V = 70$) networks, as well as relatively small (0.5) and large (1) signal to noise ratios.

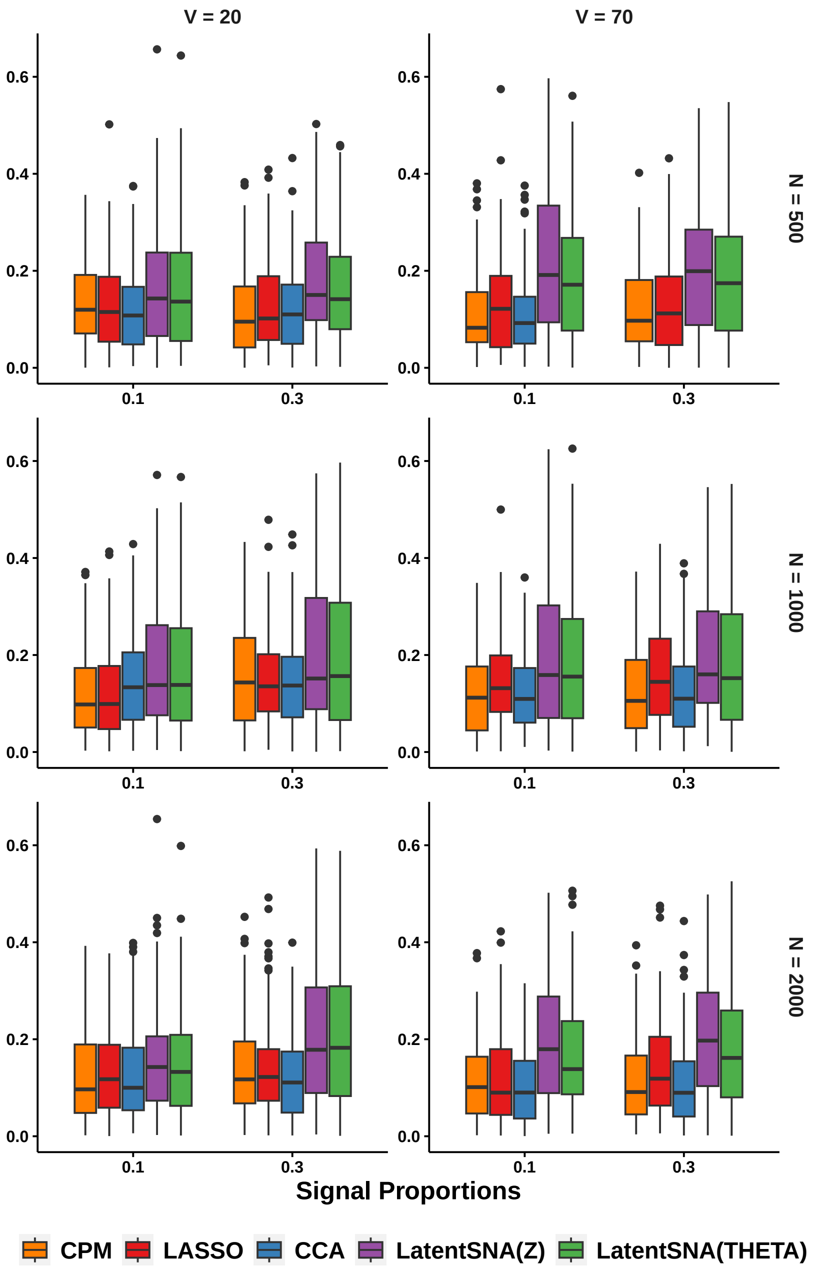

Supplementary Figure 2: The correlations between the predicted and observed psychopathology across 100 replications.

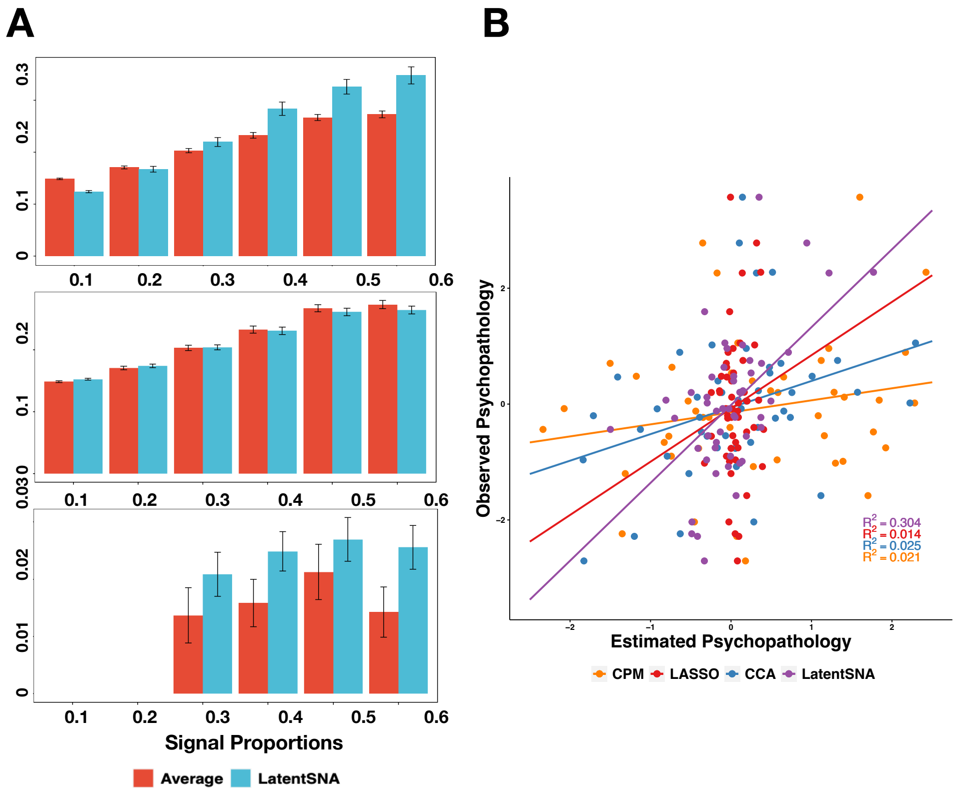

Supplementary Figure 3: LatentSNA shows satisfactory prediction accuracy for the brain connectivity and behavior. (A) The correlations between the predicted and observed connectivity across 100 replications for $N = 500$, $V = 20$, $S/N = 1$ and increasing signal proportions. (B) The scatter plot predicting behavior using latent connectivity via LatentSNA versus CPM, Lasso and CCA demonstrating the simulation results in the top right panel of Supplementary Figure 2.

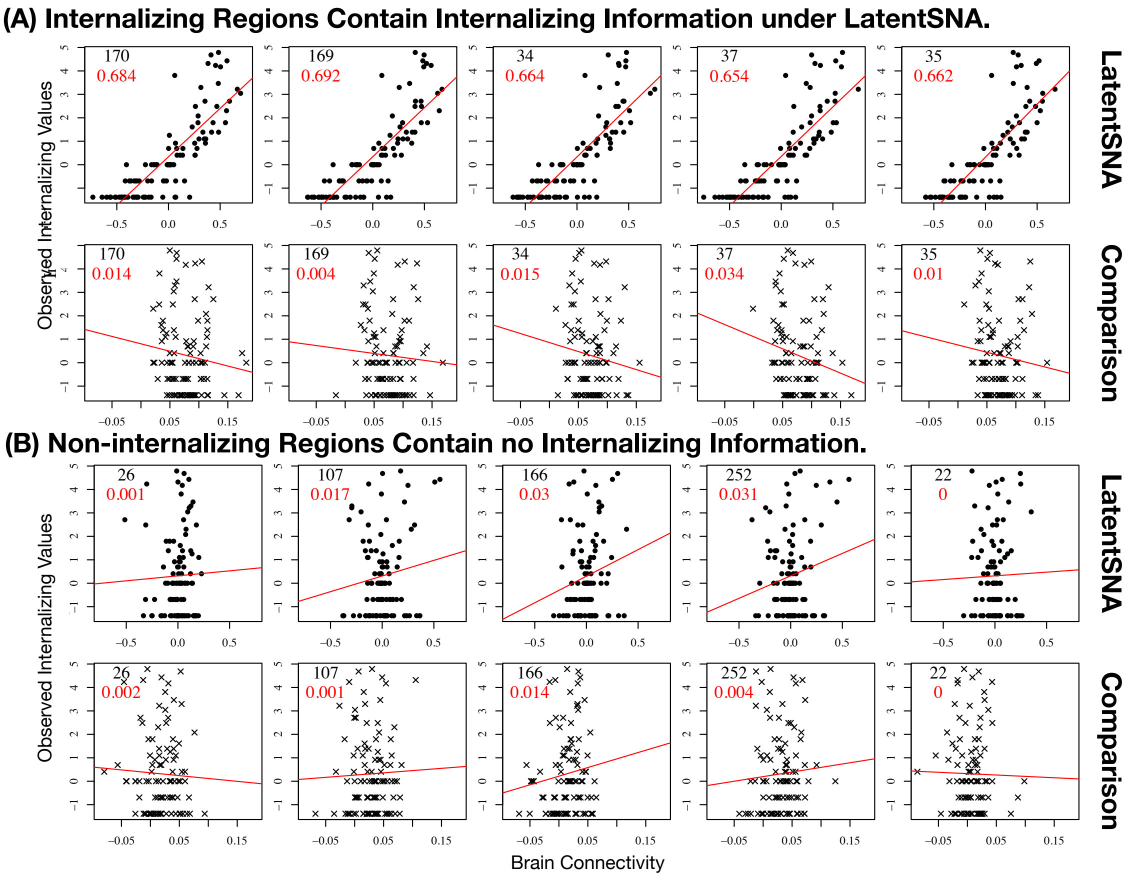

Supplementary Figure 4: The scatter plots of the observed internalizing values against the connectivity of 5 internalizing regions and 5 non-internalizing regions (region indices are shown at the top left corner in black) identified by the model. The first and third columns show the scatter plots of the observed internalizing values against the estimated latent connectivity using LatentSNA. The second and forth columns show the scatter plots of the corresponding CPM for a specific brain region, where the predictors are the sums of the significant connectivity edges based on Pearson correlations.

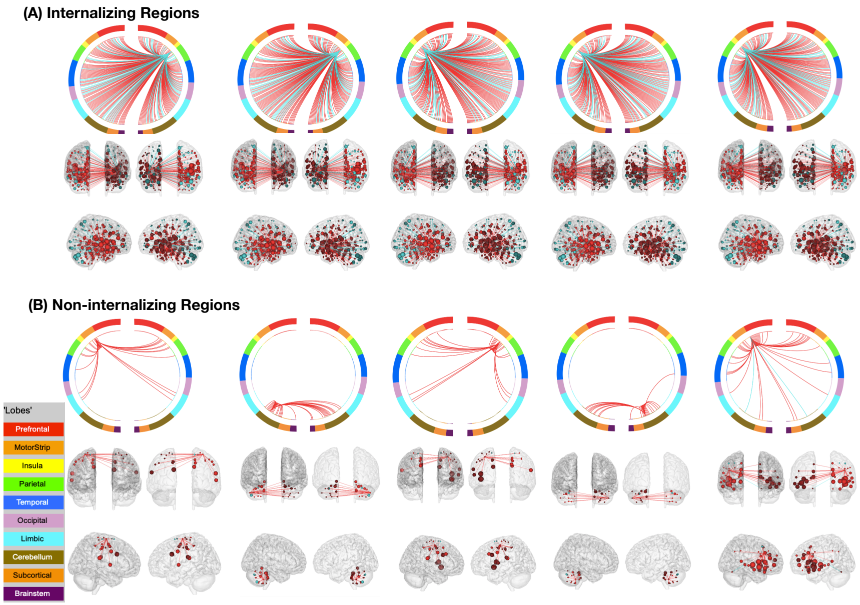

Supplementary Figure 5: The location and connectivity networks of the top 5 internalizing regions with the strongest relationships with internalizing and non-internalizing regions with no identifiable relationships with internalizing. The circle plots color groups according to anatomical locations, and the 3D brain plots show the front (top left), back (top right), right (bottom left) and left (bottom right) views.

Most regions are found with zero betweenness for both latent and observed connectivity networks (Supplementary Figure 7). With mostly zero betweenness in the functional networks, the shortest paths between nodes are direct edges as most region pairs have non-zero correlation between their time series. Regions with high strength and closeness can spread a large quantity of information efficiently through the networked brain. In the connectivity network, high strength tends to correspond with high closeness; on the low end of node strength, low strength corresponds with high closeness. The low end of node strength consists of regions with many negative edges, and these regions are close to other actors in the networks. Region 241, a region central to the visual II system is found with low node strength but high closeness and betweenness. Region 241 is a central actor in the local visual II system. It is found in the shortest paths among immediate VII regions (high betweenness) with short distances (high closeness). Meanwhile, the region has many negative connectivity edges making its overall strength low. The latent network and the observed network show similar orders of regions based on strength and closeness.

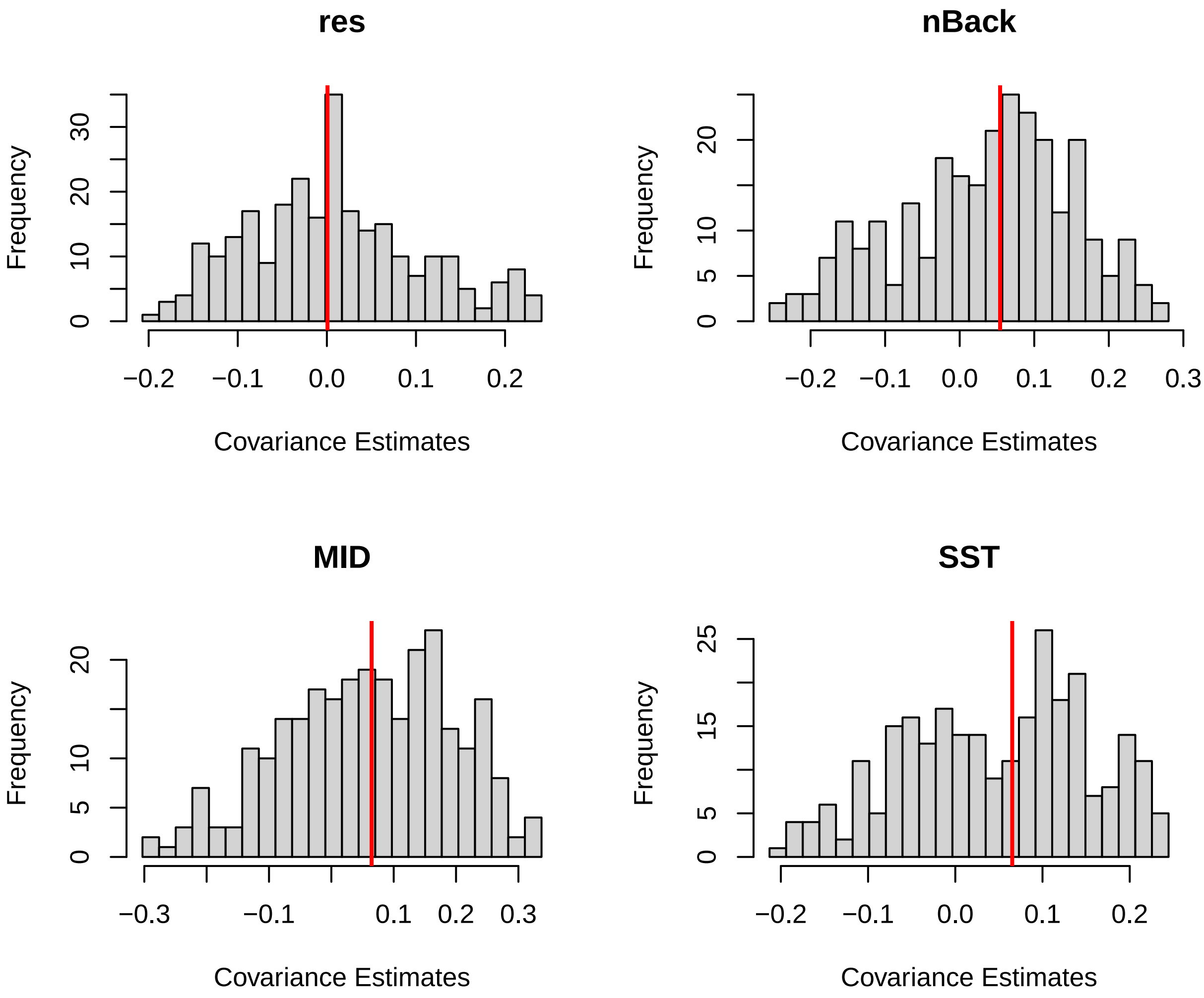

Supplementary Figure 6: The histograms of the estimated covariances between functional connectivity networks and internalizing psychopathology in the 4 conditions.

Methodology Innovation of LatentSNA

LatentSNA contributes to the current neuroimaging connectivity model literature by offering a high-power whole-brain SNA method for identifying brain-behavior links. Concentrating solely on localized effects may overlook the multifaceted nature of developing psychopathology during critical developmental stages and fail to capture substantial whole-brain changes due to widespread restructuring as the brain matures. Disjointed and disconnected connectivity biomarkers, often coupled with inconsistencies across studies and low prediction accuracy and replicability, may be attributable to low statistical power.

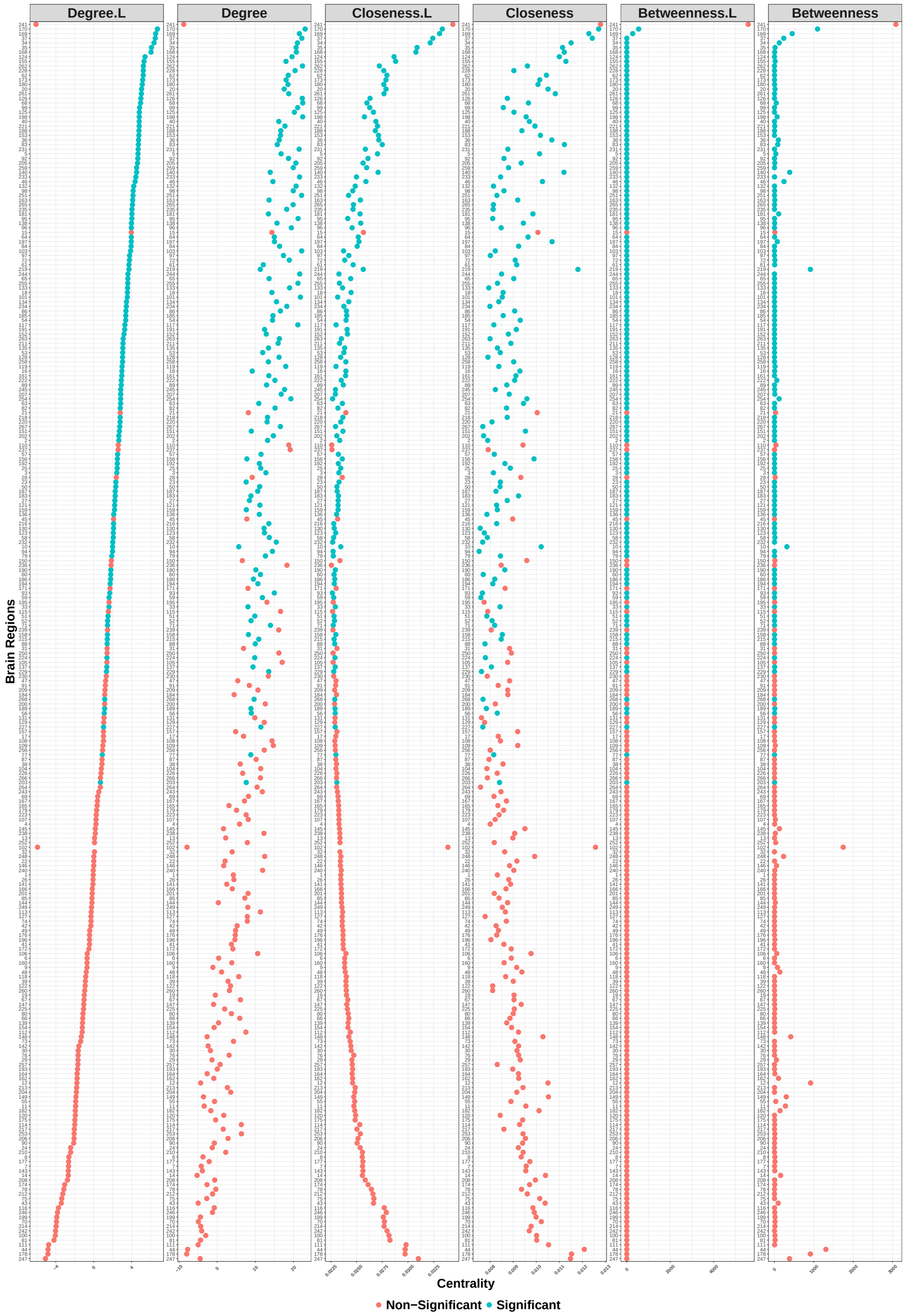

Supplementary Figure 7: The centrality of each brain regions, measured by degrees, closeness and betweenness based on the latent network (left) and the observed network (right) for an average participant during MID condition. Regions identified to play a significant role in explaining individual differences in internalizing behaviors are colored as green, and non-significant regions are colored as red.

LatentSNA addresses these limitations, improves the discovery of the exact neurobiological mechanisms underlying childhood and adolescent psychopathology and encourages the development of effective inter-

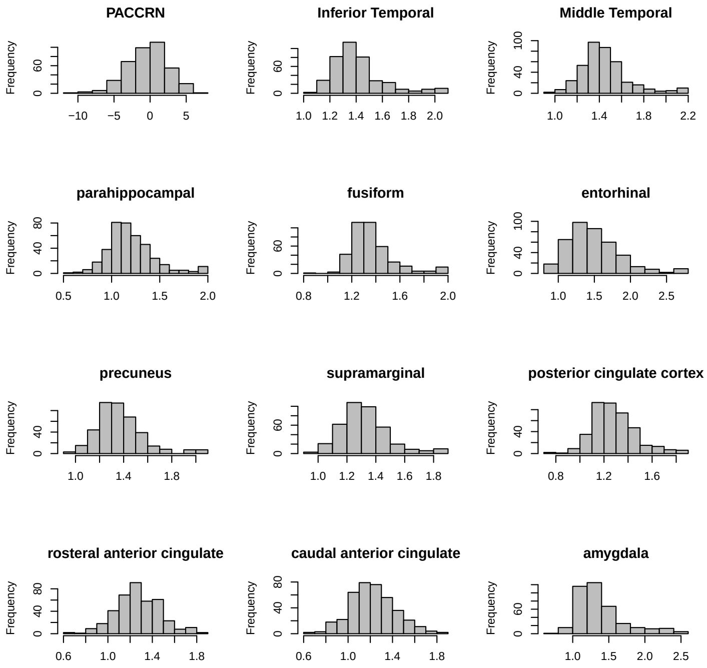

Supplementary Figure 8: Transformed Distributions of Variables

ventions.

The proposed LatentSNA uniquely contributes to the current literature of SNA methods. It is an extension of latent variables-based SNA models for imaging biomarker detection. While the current SNA methods often focus on modeling single networks, brain connectivity networks can be seen as multiplex networks with multiple layers of brain connections observed on a shared set of brain regions. To model the multiplex structures, we propose a shared set of latent variables across layers and assume a joint relational structure across sets of connectivity. We capture the individual differences in brain connectivity networks across layers and identify specific brain regions, where the covariation between layers of brain networks and the outcome variables is significant.

The LatentSNA represents an implementation of network science in a generative statistical process by innovating SNA concepts and methods for real-world networks and brain and behavior linking. In modeling objectives and approaches, the LatentSNA method is different from graphical models (GMs). A GM is used to represent joint probability distribution and construct conditional dependence between random variables . Using GMs, researchers construct a graph (network) based on whether or not two variables are

conditionally independent of each other. The edge in a brain functional network for LatentSNA represents whether/how strongly two brain regions co-activate across time; in contrast, the edge in the covariance network for GMs represents whether/how strongly two variables are conditional dependent. Crucially, the covariance networks in GMs are created out of the positive definite covariance matrices; their relationships are of a certain type, and they are more specific than the real-world observed networks including functional brain networks. In contrast to GMs, SNAs accommodate different types of real-world network structures, structures that are impossible to have for covariance networks in GMs. Therefore, GMs and SNAs are not comparable; latent variables in SNAs have different functions than latent variables in GMs.

Generalizability Results

We outline study cohorts and datasets included in the study: Alzheimer's Disease Neuroimaging Initiative Grand Opportunities and ADNI Phase 2 (ADNI-GO/2), ADNI Phase 3, Anti-Amyloid Treatment in Asymptomatic Alzheimer's Disease (A4), Human Connectome Project Aging (HCP-A), Adolescent Brain Cognitive Development Baseline (ABCD-B) and the 2-year follow-up (ABCD-2) and Transdiagnostic data collected at Yale (Supplementary Table 1). We have fitted the model to each imaging modality and outcome measure (Supplementary Table 2, 3). We focus on cognition outcomes that are often used to assess the performance of novel methods, emotion outcomes that are closely aligned with the internalizing outcomes such as depression and anxiety, disorder and focal tau PET SUVR outcomes that directly reflect biological changes in the brain.

We show the method's generalizability to detect other types of imaging biomarkers besides functional (Supplementary Table 2). We include structural imaging modalities measuring fiber density, number of fibers and fiber length (Supplementary Table 2). Note that we also included tau metrics from PET imaging from the A4 study (Supplementary Table 3). We report the average correlation between the predicted and observed outcomes in independent samples across 10 runs with the same procedure as before. The results show that using LatentSNA, we can obtain satisfactory prediction accuracy across different imaging modalities with consistent improvement over existing methods.

We show the method's generalizability to different outcomes (Supplementary Table 3). In particular, we include 4 types of outcomes with 20 outcome variables focused on cognition, disorder, emotion and focal tau PET SUVR metrics. The results show that using LatentSNA, we can obtain satisfactory prediction accuracy across different outcome measures, and we consistently improve prediction accuracy of existing methods. More specifically, we see larger improvements in predicting emotion outcomes based on fMRI data in the children's population, which corresponds with existing literature showing difficulties in phenotype prediction using children's fMRI data given the high heterogeneity and strong motion artifact. The predicability of the focal tau PET SUVR metrics is consistently higher than other types of outcomes. This result is as expected because the tau PET SUVR metrics, derived from the A4 images using post-injection images, more directly reflect brain changes than behavior outcomes.

Supplementary Table 1: Study cohorts and data

	Outcome				Neuroimaging			
Data	Cognition	Disorder	Emotion	Tau	Structural[a]	fMRI/R[b]	fMRI/T[c]	Sample Size
ADNI-GO/2	✓	✓			✓			410
ADNI-3	✓	✓				✓		174
A4	✓			✓		✓		394
HCP-Aging	✓		✓			✓		529
ABCD-B			✓			✓	✓	4,871-7,606
ABCD-2			✓			✓	✓	1,435-2,558
Transdiagnostic		✓					✓	190
Total	✓	✓	✓	✓	✓	✓	✓	8,003 - 11,861

[a]Three types of structural imaging information are used including the fiber density, the number of fibers and fiber length. [b]fMRI collected when subjects are asked to rest. [c]fMRI collected when subjects are asked to perform cognitive and emotional tasks. We include emotional n-back task (EN-back), the Stop Signal task (SST) and the Monetary Incentive Delay (MID) task conditions.

Supplementary Table 2: Prediction accuracy in independent samples with different types of imaging biomarkers

		Method							
Biomarker Type	Outcome	CPM	rCPM	GC	TNFA	SVM	RF	CNN	LatentSNA
Fiber Density	ADAS	0.223	0.256	0.238	0.265	0.279	0.330	0.279	0.646
	ECog	0.234	0.176	0.212	0.362	0.236	0.326	0.304	0.572
# Fibers	ADAS	0.226	0.381	0.692	0.297	0.258	0.373	0.381	0.692
	ECog	0.256	0.258	0.326	0.448	0.167	0.342	0.243	0.663
Fiber Length	ADAS	0.421	0.236	0.265	0.366	0.242	0.274	0.307	0.681
	ECog	0.242	0.230	0.221	0.336	0.235	0.264	0.284	0.659

Supplementary Table 3: Prediction accuracy in independent samples with different types of outcomes

				Method							
Outcome Measure	Name	Source	Condition	CPM	rCPM	GC	TNFA	SVM	RF	CNN	LSNA*
Cognition	Ecog	ADNI-3	Rest	0.245	0.301	0.304	0.341	0.366	0.315	0.299	0.551
	PACCRN	A4	Rest	0.259	0.270	0.232	0.228	0.250	0.270	0.201	0.543
	Picture Sequence Memory	HCP-Aging	Rest	0.331	0.381	0.363	0.247	0.329	0.394	0.295	0.663
	Cognition Composite			0.273	0.610	0.394	0.332	0.230	0.334	0.246	0.615
Disorder	BSI	Transdiagnostic	nBack	0.099	0.143	0.032	0.194	0.153	0.191	0.221	0.494
	ADAS	ADNI-3	Rest	0.167	0.276	0.217	0.255	0.182	0.333	0.371	0.492
Emotion	Internalizing	ABCD-2	Rest	0.091	0.200	0.102	0.267	0.118	0.149	0.149	0.897
			nBack	0.153	0.162	0.080	0.156	0.198	0.149	0.175	0.774
			MID	0.124	0.273	0.361	0.276	0.203	0.148	0.167	0.721
			SST	0.083	0.229	0.099	0.239	0.176	0.208	0.160	0.692
	Emotional Distress Depression	HCP-Aging	Rest	0.416	0.302	0.198	0.286	0.265	0.295	0.259	0.658
	PROMIS Anxiety			0.249	0.285	0.386	0.222	0.175	0.232	0.266	0.650
Tau	Inferior Temporal	A4	Rest	0.211	0.349	0.276	0.284	0.303	0.298	0.326	0.599
	Middle Temporal			0.325	0.275	0.227	0.190	0.344	0.277	0.247	0.652
	Parahippocampal			0.254	0.141	0.229	0.222	0.152	0.280	0.276	0.624
	Fusiform			0.366	0.206	0.314	0.428	0.320	0.288	0.340	0.663
	Entorhinal			0.274	0.242	0.299	0.348	0.232	0.146	0.200	0.642
	Precuneus			0.303	0.208	0.271	0.369	0.278	0.411	0.300	0.653
	Supramarginal			0.327	0.317	0.288	0.299	0.380	0.222	0.306	0.613
	Posterior cingulate cortex			0.298	0.354	0.290	0.176	0.159	0.354	0.303	0.588
	Rosteral anterior cingulate			0.254	0.275	0.271	0.236	0.249	0.297	0.384	0.611
	Caudal anterior cingulate			0.270	0.274	0.225	0.243	0.301	0.295	0.317	0.569
	Amygdala			0.252	0.200	0.170	0.263	0.303	0.236	0.231	0.588
