# Supplementary material for: Latent space-based network analysis for brain–behavior linking in neuroimaging
Source: Nat Methods. 2025 Dec 4;23(1):225–35. doi: 10.1038/s41592-025-02896-9 (PMC13002467; doi:10.1038/s41592-025-02896-9)
Supplement: Supplementary file 2 — Reporting Summary [file 41592_2025_2896_MOESM2_ESM.pdf]

nature portfolio

Corresponding author(s): Selena Wang

Last updated by author(s): May 30, 2025

Reporting Summary

Nature Portfolio wishes to improve the reproducibility of the work that we publish. This form provides structure for consistency and transparency in reporting. For further information on Nature Portfolio policies, see our Editorial Policies and the Editorial Policy Checklist.

Statistics

For all statistical analyses, confirm that the following items are present in the figure legend, table legend, main text, or Methods section.

n/a	Confirmed	
☐	☒	The exact sample size (*n*) for each experimental group/condition, given as a discrete number and unit of measurement
☐	☒	A statement on whether measurements were taken from distinct samples or whether the same sample was measured repeatedly
☐	☒	The statistical test(s) used AND whether they are one- or two-sided<br>*Only common tests should be described solely by name; describe more complex techniques in the Methods section.*
☐	☒	A description of all covariates tested
☐	☒	A description of any assumptions or corrections, such as tests of normality and adjustment for multiple comparisons
☐	☒	A full description of the statistical parameters including central tendency (e.g. means) or other basic estimates (e.g. regression coefficient) AND variation (e.g. standard deviation) or associated estimates of uncertainty (e.g. confidence intervals)
☐	☒	For null hypothesis testing, the test statistic (e.g. *F*, *t*, *r*) with confidence intervals, effect sizes, degrees of freedom and *P* value noted<br>*Give P values as exact values whenever suitable.*
☐	☒	For Bayesian analysis, information on the choice of priors and Markov chain Monte Carlo settings
☐	☒	For hierarchical and complex designs, identification of the appropriate level for tests and full reporting of outcomes
☐	☒	Estimates of effect sizes (e.g. Cohen's *d*, Pearson's *r*), indicating how they were calculated

Our web collection on statistics for biologists contains articles on many of the points above.

Software and code

Policy information about availability of computer code

Data collection	Public data from the ABCD study, no new data are collected
Data analysis	R 4.2.0 can be downloaded at https://cran.r-project.org/bin/windows/base/old/4.2.0/<br>The code has been released on the code ocean with digital object identifier (DOI) 10.24433/CO.8871706.v1. Code is also available here: https://github.com/selenashuowang/latentSNA

For manuscripts utilizing custom algorithms or software that are central to the research but not yet described in published literature, software must be made available to editors and reviewers. We strongly encourage code deposition in a community repository (e.g. GitHub). See the Nature Portfolio guidelines for submitting code & software for further information.

Data

Policy information about availability of data

All manuscripts must include a data availability statement. This statement should provide the following information, where applicable:

- Accession codes, unique identifiers, or web links for publicly available datasets
- A description of any restrictions on data availability
- For clinical datasets or third party data, please ensure that the statement adheres to our policy

We used brain imaging data from the first release of the ABCD study collected from 11, 875 children aged between 9 to 10 years old. Public access: https://abcdstudy.org/

Human research participants

Policy information about studies involving human research participants and Sex and Gender in Research.

Reporting on sex and gender	We use functional brain imaging data from the first release of the Adolescent Brain Cognitive Development (ABCD) study. And We have 6,185 males and 5,681 females.
Population characteristics	We used brain imaging data from the first release of the ABCD study collected from 11, 875 children aged between 9 to 10 years old. The blood-oxygen-level-dependent (BOLD) functional activation was recorded for children during resting state (RS) and when they performed three emotional and cognitive tasks.<br>Internalizing psychopathology represents a spectrum of conditions characterized by negative emotion including depression, anxiety and phobias. In the ABCD study, the internalizing psychopathology is collected via self-reported survey using the Child Behavior Checklist (CBCL, Stavropoulos et al. , 2017), which includes 119 items aggregated into 8 empirical sub-scales. Three sub-scales of CBCL, anxious-depressed (13 items), withdrawn-depressed (8 items) and somatic complaints (11 items) are parts of the internalizing psychopathology. The multivariate representation of the internalizing psychopathology with anxious-depressed, withdrawn-depressed and somatic complaints variables likely outperforms the univariate representation (sum of the three variables) due to the loss of information in the latter.
Recruitment	We used brain imaging public data from the first release of the ABCD study collected from 11, 875 children aged between 9 to 10 years old.
Ethics oversight	Adolescent Brain Cognitive Development(ABCD) study

Note that full information on the approval of the study protocol must also be provided in the manuscript.

Field-specific reporting

Please select the one below that is the best fit for your research. If you are not sure, read the appropriate sections before making your selection.

☒ Life sciences ☐ Behavioural & social sciences ☐ Ecological, evolutionary & environmental sciences

For a reference copy of the document with all sections, see nature.com/documents/nr-reporting-summary-flat.pdf

Life sciences study design

All studies must disclose on these points even when the disclosure is negative.

Sample size	We use functional brain imaging data from the first release of the Adolescent Brain Cognitive Development (ABCD) study, collected from 11, 875 children aged between 9 to 10 years old (Casey et al, 2018). The functional MRI (fMRI) data is collected from children when they were resting capturing intrinsic brain activity (Rest), when they were performing the the emotional n-back task (EN-back), the Stop Signal task (SST), and the Monetary Incentive Delay (MID) task.
Data exclusions	We included 7, 606 adolescents with RS functional connectivity capturing intrinsic brain functional activity. We separately investigated the functional connectivity of 4, 871 adolescents who are asked to perform the emotional n-back task (EN-back), 5, 096 adolescents who are asked to perform the Stop Signal task (SST) and 5, 298 adolescents who are asked to perform the Monetary Incentive Delay (MID) task.
Replication	Results are successfully replicated with different task conditions.
Randomization	N/A; there were no experimental groups in the study.
Blinding	N/A; there was no group allocation in the study.

Reporting for specific materials, systems and methods

We require information from authors about some types of materials, experimental systems and methods used in many studies. Here, indicate whether each material, system or method listed is relevant to your study. If you are not sure if a list item applies to your research, read the appropriate section before selecting a response.

Materials & experimental systems

n/a	Involved in the study
☒	☐ Antibodies
☒	☐ Eukaryotic cell lines
☒	☐ Palaeontology and archaeology
☒	☐ Animals and other organisms
☒	☐ Clinical data
☒	☐ Dual use research of concern

Methods

n/a	Involved in the study
☒	☐ ChIP-seq
☒	☐ Flow cytometry
☐	☒ MRI-based neuroimaging

Magnetic resonance imaging

Experimental design

Design type

resting state and and different cognitive, emotional and behavioral task states

Design specifications

We use functional brain imaging data from the first and second releases of the Adolescent Brain Cognitive Development (ABCD) study, collected from 11, 875 children aged between 9 to 10 years old (Casey et al, 2018).

Behavioral performance measures

Internalizing psychopathology data represents a spectrum of conditions characterized by negative emotion including depression, anxiety and phobias. In the ABCD study, the internalizing psychopathology is collected via self-reported survey using the Child Behavior Checklist (CBCL, Stavropoulos et al. , 2017), which includes 119 items aggregated into 8 empirical sub-scales. Three sub-scales of CBCL, anxious-depressed (13 items), withdrawn-depressed (8 items) and somatic complaints (11 items) are parts of the internalizing psychopathology. The multivariate representation of the internalizing psychopathology with anxious-depressed, withdrawn-depressed and somatic complaints variables likely outperforms the univariate representation (sum of the three variables) due to the loss of information in the latter.

Acquisition

Imaging type(s)

functional

Field strength

3T

Sequence & imaging parameters

High spatial and temporal resolution multiband echo-planar imaging (EPI) resting-state fMRI data with fast integrated distortion correction are acquired using three 3T scanner platforms: Siemens Prisma, General Electric (GE) 750, and Phillips. Resting-state fMRI parameters are similar across platforms: a standard multiband EPI sequence, repetition timeTR)/echo time (TE) = 800/30 ms, voxel spacing size = 2.4 x 2.4 x 2.4 mm, slice number = 60, flip angle (FA) = 52, field oiview (FOV) = 216 x 216 mm, multiband acceleration = 6.
ABCD_Website_MRI_Acq link: https://abcdstudy.org/wp-content/uploads/2021/05/ABCD_Website_MRI_Acq.pdf

Area of acquisition

Whole brain scans were acquired.

Diffusion MRI ☐ Used ☒ Not used

Preprocessing

Preprocessing software

BioImage Suite and SPM5.

Normalization

We removed the linear trend from all signals in accordance with the methodologydetailed in Shen et al. (2013).

Normalization template

MNI

Noise and artifact removal

We deleted scans with more than 0.10 mm mean frame-to-frame displacement.

Volume censoring

First, we performed motion correction and slice-time correction using SPM5; and via BioImage Suite, the data were registered to a standardized 3mm X 3mm x 3mm common space, where we generated masks representing white matter, gray matter, and cerebrospinal fluid (CSF) and computed the mean time courses for both white matter and CSF. We orthogonalized each gray matter time course with respect to the mean time courses of both white matter and CSF, and we orthogonalized each gray matter timec ourse to the six motion-related signals via SPM5. We then applied a bandpass Butterworth filterwith a frequency range of 0.02Hz to 0.1Hz to the orthogonalized time courses. We used a Gaussiankernel with a full-width at half-maximum (FWHM) of 6mm to enhance spatial coherence and spatialsmoothing. Lastly, we removed the linear trend from all signals in accordance with the methodologydetailed in Shen et al. (2013). We deleted scans with more than 0.10 mm mean frame-to-framedisplacement. Additional details about the standard preprocessing procedures, such as slice timeand motion correction, registration to the MNI template can be found in Greene et al 2018) andHorien et al (2019).

Statistical modeling & inference

Model type and settings	Imaging Biomarker effects described in the method section. It is built on joint bayesian framework
Effect(s) tested	Co-variation between functional connectivity and internalizing

Specify type of analysis: ☒ Whole brain ☐ ROI-based ☐ Both

Statistic type for inference (See Eklund et al. 2016)	New method is proposed to test region-specific co-variation in whole functional connectivity and internalizing
Correction	Inference under Bayesian posterior sampling

Models & analysis

n/a	Involved in the study
☐	☒ Functional and/or effective connectivity
☐	☒ Graph analysis
☐	☒ Multivariate modeling or predictive analysis

Functional and/or effective connectivity	pearson correlation
Graph analysis	Joint modeling framework with both connectivity and behavior as dependent variables, statistical network analysis is used for modeling graphs
Multivariate modeling and predictive analysis	Statistical network analysis is used to reduce dimension via latent variable modeling. Predictions of future behaviors and connectivity are performed under the joint modeling framework.
